# Role of *MYOC* and *OPTN* sequence variations in Spanish patients with Primary Open-Angle Glaucoma

**Published:** 2007-06-14

**Authors:** Francisco López-Martínez, María-Pilar López-Garrido, Francisco Sánchez-Sánchez, Ezequiel Campos-Mollo, Miguel Coca-Prados, Julio Escribano

**Affiliations:** 1 Servicio de Oftalmología, Complejo Hospitalario Universitario de Albacete (Hospital Perpetuo Socorro), Albacete, Spain; 2 Área de Génetica, Facultad de Medicina/Centro Regional de Investigaciones Biomédicas (CRIB), Albacete, Spain; 3 Servicio de Oftalmología, Hospital Virgen de los Lirios, Alicante, Spain; 4 Department of Ophthalmology and Visual Science, Yale University School of Medicine, New Haven, CT

## Abstract

**Purpose:**

To retrospectively investigate the contribution of *myocilin* (*MYOC*) and *optineurin* (*OPTN*) sequence variations to adult-onset ocular hypertension (OHT) and primary open-angle glaucoma (POAG) in Spanish patients.

**Methods:**

The promoter region and the three exons of *MYOC* were analyzed by direct PCR DNA sequencing in 40 OHT and 110 POAG unrelated patients. We used 98 subjects in whom OHT or glaucoma had been ruled out as controls. We also screened the complete coding region of the *OPTN* gene (exons 4-16) in all subjects by single-stranded conformational polymorphisms (SSCPs).

**Results:**

We identified six common single nucleotide polymorphisms (SNPs) in the promoter region of *MYOC* (-1000C>G, -387C>T, -306G>A, -224T>C, -126T>C and -83G>A) and a polymorphic GT microsatellite (-339(GT)11-19). In addition, we detected four novel, rare DNA polymorphisms. None of these DNA sequence variations were associated with either OHT or POAG. We also found three (2.7%) POAG patients with *MYOC* pathogenic mutations. Two of these pathogenic mutations (Gln368Stop and Ala445Val) were previously described whereas the third (Tyr479His) was novel. Transient expression of the novel mutation in 293T cells supported its pathogenicity. Only two *OPTN* polymorphisms, which are not associated with the disease, were detected.

**Conclusions:**

Overall, our data show that in Spain a minority of adult-onset high-pressure POAG patients carry heterozygous disease-causing mutations in the *MYOC* gene and that *OPTN* is not involved in either OHT or POAG.

## Introduction

Glaucoma is a complex and genetically heterogeneous disease characterized by the progressive apoptotic death of retinal ganglion cells. This process leads to the excavation of the optic nerve head and to progressive and irreversible visual field loss [1,2]. Glaucoma is the second leading cause of blindness with prevalence of 0.15% in the total population and of approximately 2-4% among the population over the age of 40. Primary open-angle glaucoma (POAG) is the most common form of glaucoma, that manifests as an insidious and chronic condition characterized by a gonioscopically open angle. Although most people will not develop glaucomatous damage despite having an intraocular pressure (IOP) well above 21 mmHg, elevated IOP (>21 mmHg), originated by an increase in aqueous outflow resistance, is the most important risk factor in glaucoma [3]. It is speculated that elevated IOP could compress the optic nerve at the lamina cribosa. Depending on individual susceptibility factors, elevated IOP might damage ganglion cell axons and local glial cells as well as impair the capillary blood supply to the region. These events could progressively lead to the apoptotic death of ganglion cells [4]. Other risk factors include age, gender, myopia, and vascular and genetic factors. It has also been reported that changes in expression of genes such as p21(WAF1/CIP1) and 14-3-3 sigma may indicate an increased risk for glaucoma [5].

Genetically, POAG shows a complex pattern of inheritance with sporadic manifestations in most patients. The *myocilin* (*MYOC*) gene is mutated in 3-5% of sporadic patients in populations around the world [6-10]. This gene is composed of three exons and is ubiquitously expressed in many human tissues including the iris, ciliary body, and trabecular meshwork (TM) [11-13]. The majority of *MYOC* disease-causing mutations map to the olfactomedin-like domain of the protein, which is encoded by exon 3 [12]. In addition, heterozygous mutations in cytochrome P450 1B1 *CYP1B1*, a gene involved in primary congenital glaucoma, have been identified in 4-9% of affected POAG subjects from France [14,15], India [16], and Spain [17].

The *optineurin* (*OPTN*) gene consists of sixteen exons and the first three are noncoding. The *OPTN* gene is expressed in ocular tissues such as retina, TM, and nonpigmented ciliary epithelium [18]. Mutations in this gene predominately result in normal tension glaucoma [18], a subtype of glaucoma featured by normal IOP, but its role in high-pressure POAG is still controversial.

We report the first complete mutational analysis of the promoter and coding regions of *MYOC* and the coding region of the *OPTN* gene in Spanish patients diagnosed with adult-onset POAG. We found in this population, disease-causing mutations in the olfactomedin-like domain, encoded by the third exon of *MYOC*, are present in 2.7% of sporadic POAG cases. Our data also enable us to rule out a role of *OPTN* sequence variations in the development of POAG in Spanish patients.

## Methods

### Subjects

One hundred and ten unrelated native Spanish patients diagnosed with POAG and forty diagnosed with OHT, were studied retrospectively for *MYOC* and *OPTN* mutations. The control group was composed of 98 individuals in whom glaucoma was ruled out. All the individuals were recruited in the Department of Ophthalmology, University Hospital of Albacete, Spain ("Servicio de Oftalmología, Complejo Hospitalario Universitario de Albacete").

The following conditions were required to diagnose POAG: exclusion of secondary causes (e.g., trauma, uveitis, steroid-induced or neovascular glaucoma); open anterior chamber angle (grade III-IV gonioscopy); IOP higher than 21 mmHg; characteristic optic disc changes; and an alteration of the visual field, tested by automated perimetry (with Humphrey's perimeter). The global indices such as mean deviation (MD) and pattern standard deviation (PSD) of the baseline visual fields were analyzed for all cases. All study subjects underwent a complete ocular examination. The study protocol was approved by the Ethics Committee for Human Research of the University Hospital of Albacete and followed the tenets of the Declaration of Helsinki. Informed consents were obtained from all the study subjects.

Patients were classified as having early (MD better than -6 dB), moderate (MD between -6 and -12 dB), or severe (MD worse than -12 dB) visual field alteration according to the classification by Hodapp et al. [19]. Medical treatment primarily included topical beta-blockers and prostaglandin analogues.

### Sequence variation screening

Genomic DNA was extracted from the peripheral leukocytes of all studied subjects with the Perfect gDNA Blood Mini kit (Eppendorf, Madrid, Spain) according to the manufacturer's protocol. The promoter (nucleotides -1 to -1117) and the three exons of *MYOC* were amplified using primers designed to allow analysis of splicing consensus sequences (Table 1). PCRs were performed in a 50 μl volume containing 50-100 ng of genomic DNA, 10 pmol of forward and reverse primers, 2 mM MgCl_2_ for exons 1 and 3, 0.5 mM MgCl_2_ for exon 2, 100 μM of each dNTP, and 1 U of Taq DNA polymerase (Biotools, B&M Labs, Madrid, Spain). Thermocycling included an initial denaturation step at 94 °C for four min followed by 35 cycles of denaturation, annealing, and extension (Table 1). A final cycle was performed at 72 °C for seven min. Terminator cycle sequencing was carried out using the BigDye® (v3.1) kit (Applied Biosystems, Foster City, CA). The products of sequencing reactions were analysed in an automated capillary DNA sequencer (ABI Prism 3100-Avant genetic analyzer; Applied Biosystems).

**Table 1:** Oligonucleotide primers used for amplification of the *myocilin* gene.

**Region**	**Primer sequence (5'-3')**	**Annealing temperature and time (°C/s)**	**Extension time (s)**	**Amplicon length (bp)**
Promoter	F: TCCAGAAAGCCTGTGAATTTGA			
	R: AGGCAGGCCAGAAGCAGC	61.5/20	60	1117
Exon I	F: CTCACCAAGCCTCTGCAATG			
	R: TGAACTCAGAGTCCCCCCAC	62/20	15	654
Exon II	F: ACATAGTCAATCCTTGGGCC			
	R: CATGAATAAAGACCATGTGG	55/20	20	239
Exon III	F: TCTGTGTTTGGAAAGATTATGG			
	R: CCTGAGCATCTCCTTCTGCC	59/30	40	890

Annealing temperature and time, extension time for each primer pair and the predicted length for each of the PCR DNA products in base pairs (bp) are listed. In the Primer sequence column, F indicates forward and R indicates reverse.

### Single stranded conformational polymorphism analysis

Mutations in the 13 coding exons (4-16) of the *OPTN* gene were screened by PCR-SSCP. Each exon was amplified by PCR in 50 μl reaction volumes using the primers, annealing temperatures, and times detailed in Table 2. Primers were also designed to allow analysis of splicing consensus sequences. Each reaction contained 2.0 mM MgCl_2_, 10 pmol of forward and reverse primers, 100-200 μM dNTPs, 0.5 U Taq Polymerase (Biotools), and 50-100 ng of genomic DNA. Reactions were denatured at 94 °C for four min followed by 35 cycles of denaturation, annealing, and extension (Table 2) as well as a final extension of 72 °C for seven min. PCR products (2-4 μl) were added to two volumes (4-8 μl) of SSCP stop solution consisting of 95% deionized formamide, 10 mM EDTA, 1 mg/ml Bromophenol blue, 1 mg/ml Xylene Cyanol (all these reagents were supplied by Sigma-Aldrich, St. Louis, MO), were denatured at 95 °C for ten min, and were chilled on ice for five min. The presence of abnormally migrating bands was confirmed by three different electrophoretic conditions using acrylamide gels (Table 3). Electrophoresis was performed on a DCode^TM^ Universal Mutation Detection System (Bio-Rad, Hercules, CA) in 0.5X TBE buffer (45 mM Tris, 45 mM boric acid, 1 mM EDTA). After the run, gels were removed from the apparatus and the DNA bands were visualized by silver staining [20]. Mobility shift of single-strand DNA from the normal pattern indicated the presence of a possible mutation and was further investigated by sequence analysis of genomic DNA.

**Table 2:** Oligonucleotide primers used for amplification of the *optineurin* gene.

**Exon**	**Primer sequence (5'-3')**	**Annealing temperature and time (C/s)**	**Extension time (s)**	**Amplicon length (bp)**
4	F: GCCAGTGGG TTTGTGGGAC			
	R: TGCAAAGGGATGGCATTTC	60/20	20	320
5	F: CACTTTCCTGGTGTGTGACTCC			
	R: AAACAACATCACAATGGATCG	60/25	20	281
6	F: CCCAGCCTTAGTTTGATCTG			
	R: GGGGAGGCTTTATAGTTTGC	60/20	20	278
7	F: CATATTGTGTTAAATCCCTTGC			
	R: GGTCACAACATTTGACCTCC	60/20	20	198
8	F: AGTCTTTGGAATTTTTCTGATG			
	R: ATGGGTGAACTGTATGGTATC	60/30	30	287
9	F: GCTATTTCTCTTAAAGCCAAAG			
	R: ACTCTCGTGTGTGTGGGTG	60/30	14	205
10	F: GAGGTTTGTTTAATGTCAGATG			
	R: TCAAAGGAGGATAAAATTGC	55/30	30	211
11	F: CACTGCGACGTAAAGGAGC			
	R: GCTGCCCTTCTGACTCAAC	65/30	30	231
12	F: ATATTTTCCCCAGGATTCC			
	R: AACGTTCAACAGTTTCTGTTC	55/30	30	196
13	F: CAGGCAGAATTATTTCAAAAC			
	R: AATACAGTCAGGGCTGGC	60/30	5	260
14	F: ACAGCACTACCTCCTCATCGC			
	R: GATGTGAGCTCTGGGTCCTCC	65/30	20	231
15	F: TCAGTGTTGTCATGTTTCGGG			
	R: TGAAAATCCAGGATCACACG	60/30	30	171
16	F: CCTGCAAAATGGAACTAATGG			
	R: ACATTTACCAACAGTTTTGGG	61/20	15	203

Annealing temperature and time, extension time for each primer pair and the predicted length for each of the PCR DNA products in base pairs (bp) are listed. In the Primer sequence column, F indicates forward and R indicates reverse.

**Table 3:** Electrophoretic conditions used for single-stranded conformational polymorphism analysis.

**Acrylamide/bisacrylamide (10%)**	**Glycerol (%)**	**Temperature**	**Voltage (V)**
49/1	0	RT	300
29/1	5	RT	300
29/1	0	4	600

Acrylamide/bisacrylamide and glycerol composition of the gels as well as temperature and voltage used for electrophoresis are indicated.

### Linkage disequilibrium and haplotype construction

Pairwise linkage disequilibrium (LD) between the SNPs with minor allele frequencies (MAF) higher than 5% was measured as D' [21] using the Haploview software version 3.2 [22]. Regions of strong LD (LD blocks) were inferred using the confidence-interval model proposed by Gabriel and colleagues [23] as implemented in Haploview. Haplotype reconstruction was done with the expectation-maximization algorithm in PowerMarker v. 3.22 [24].

### Statistical analysis

The significance of the difference in frequencies of DNA polymorphisms between patients and control subjects was determined by the x^2^ test when all expected values were five or more. The Fisher's exact test was used when expected values were less than five. Data were statistically treated by using the SigmaStat 2.0 software (SPSS Science, Inc., Chicago, IL).

### Expression of mutant myocilin in 293T cells

Myocilin point mutations were obtained as previously described [25]. The specific PCR primers used for mutagenesis were: 5'-CCA GAA CTG TCA TAA CAT ATG AGC TGA ATA CC-3' (forward) and 5'-GGT ATT CAG CTC ATA TGT TAT GAC AGT TCT GG-3' (reverse) for Arg346Thr; 5'-CAG CAG CAT GAT TGA CCA CAA CCC CCT GGA GAA G-3' (forward) and 5'-CTT CTC CAG GGG GTT GTG GTC AAT CAT GCT GCT G-3' (reverse) for Tyr479His.

Human embryonic kidney 293T cells were bought from the American Type Culture Collection (ATCC, Rockville, MD). Transient expression of wild-type and mutant myocilins was performed as described [25]. An expression analysis of the different myocilin forms was performed by western immunoblot using an anti-myc antibody (9E10, Santa Cruz, Valencia, CA) diluted at 1:400-1:500 [25]. Fluorescence microscopy was also carried out as described [25].

## Results

### Phenotype of patients

A total of 110 unrelated and sporadic POAG patients were studied. In addition, we analyzed 40 cases diagnosed with OHT. The control group included 98 individuals in whom glaucoma was ruled out. The main clinical features of most of these subjects have been reported in a previous study of *CYP1B1* mutations in Spanish patients with POAG [17]. Subjects with mutations in the *CYP1B1* gene were not included in the present study. The three groups were homogeneous with respect to gender and age (p>0.1; Table 4). Patients were under medical treatment to reduce IOP. Therefore, their IOP mean values were below 21 mmHg at the time of the study (Table 4), which indicated that treatment was effective. The mean IOP and C/D ratios in both eyes of glaucoma patients were significantly higher (p<0.01) than in controls (Table 4). The visual field status of the eyes from POAG patients was severe for 12.7%, moderate for 27.4%, early for 49.6%, and normal for 7.3%. The visual field could not be determined in 3.0% of eyes. Normal eyes were from patients who showed monolateral visual field alterations. The visual field was normal in the OHT patients.

**Table 4:** Clinical characteristics of participants in the study.

**Variable**	**OHT (n=40)**	**POAG (n=110)**	**Control (n=98)**
Age (mean±SD)	56.5±11.9	62.2±11.4	61.1±12.6
Female (%)	47.5	48.2	51.1
Male (%)	52.5	51.8	48.9
IOP OD (mean±SD)	17.6±4.0	17.7±3.2	15.1±3.1
IOP OS (mean±SD)	17.8±4.4	17.3±3.0	15.2±3.1
C/D OD (mean±SD)	0.3±0.2	0.5±0.2	0.2±0.2
C/D OS (mean±SD)	0.3±0.2	0.5±0.2	0.2±0.2

Details of clinical features of 40 OHT, 110 POAG patients, and 98 unaffected controls are shown in the table. Since this is a retrospective study, IOP values before medical treatment were generally not available. C/D indicates cup-disc ratio of optic nerve; OD indicates right eye; OS indicates left eye; and SD indicates standard deviation. The three groups were homogeneous with respect to gender and age. Mean IOP and C/D ratios in glaucoma patients were higher than in controls. In the "Variable" column, the listed age was at the time of the study.

### Identification of *myocilin* sequence variations and genotype-phenotype correlation

**Figure 1:**
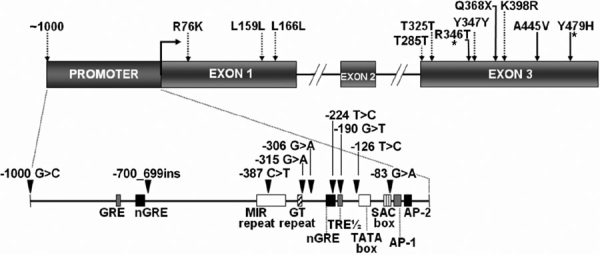
Genomic structure of the human *myocilin* gene and location of identified DNA sequence variations. The promoter and the three exons are represented by boxes. Consensus regulatory sequences in the promoter region are depicted in the inset. Pathogenic mutations and polymorphisms in the coding region are indicated by solid and dashed arrows, respectively. Sequence variations in the promoter region are shown by arrowheads. Novel mutations are shown by asterisks. All mutations are defined in terms of the one-letter code. Designated features include AP-1-like and AP-2-like sequences, putative TATA and SAC boxes, glucocorticoid response element (GRE), negative glucocorticoid response element (nGRE), thyroid response element (TRE) and a MIR repeat [26,63]. -700_699ins: -700_699insCAGACACACATATACATGCACATACACA.

Genomic DNA from each of 110 POAG and 40 OHT unrelated Spanish patients was screened by direct PCR sequencing for mutations in the promoter (nucleotides -1 to -1117) and in the three exons including consensus splicing sequences of the *MYOC* gene. OHT patients were investigated because elevated IOP is one of the major risk factors for the development of glaucoma and in our group of POAG patients, OHT was the first stage of the disease. The same genetic analysis was performed in 98 control subjects. Allele and genotype frequencies for all sequence variations were calculated. Genotype frequencies did not deviate from the Hardy-Weinberg equilibrium (data not shown). We identified six common SNPs (MAF >5% in at least one group) in the promoter region: -1000C>G, -387C>T, -306G>A, -224T>C, -126T>C, and -83G>A (Figure 1 and Table 5). Two of them were located close to two consensus sequences: SNP -224T>C was mapped next to the 3' end of one negative glucocorticoid response element (nGRE) and SNP -83G>A was placed at the 3' end of a SAC box (Figure 1). The two consensus sequences are putatively involved in the regulation of myocilin expression. The promoter polymorphism, -387C>T, was located in a mammalian interspersed repeat (MIR) element [26]. We also detected a polymorphic GT microsatellite at position -339 (Figure 1) with seven alleles ranging from eleven to nineteen repetitions. Alleles 11, 16, and 19 presented the lowest frequencies in the three groups of subjects (0-1.6%) while allele 13 was the most frequent, ranging from 34.1% in POAG patients to 40.8% in controls (Table 5). Alleles 17 and 18 were not detected in our population. The genotype 13/15 was highly represented in the three groups and varied from 23.6% in POAG to 33.3% in OHT subjects (Table 6). This polymorphism has been previously described in Chinese [27] and Swedish [28] populations. We did not detect any statistically significant association between these *MYOC* promoter polymorphisms and either POAG or OHT (Table 5 and Table 6). We also observed the common coding SNP, Arg76Lys, in this population (Figure 1) with similar allele and genotype frequencies in cases and controls (Table 5 and Table 6).

**Table 5:** Allele frequencies of *myocilin* promoter and coding sequence variations in primary open-angle glaucoma, adult-onset ocular hypertension, and control subjects.

**Polymorphism**	**Allele**	**POAG (%) (n=220)**	**OHT (%) (n=80)**	**Control (%) (n=196)**	P*	**P#**
-1000C>G	C	90	88.7	86.7	0.29	0.64
	G	10	11.3	13.3		
-700_699ins	WT	100	99.5	100	1	0.28
	I	0	0.5	0		
-387C>T	C	89.5	88.7	86.7	0.37	0.64
	T	10.5	11.3	13.3		
-339(GT)11-19	11	0.5	0	0	0.14	0.48
	12	9.5	16.6	16.3		
	13	34.1	40	40.8		
	14	27.3	15	19.4		
	15	27.3	26.7	22.4		
	16	1.3	0	1		
	19	0	1.6	0		
-315G>A	G	100	99.5	100	1	0.28
	A	0	0.5	0		
-306G>A	G	80.5	85	81.6	0.8	0.53
	A	19.5	15	18.4		
-224T>C	T	80	71.2	69.9	0.06	0.8
	C	20	28.8	30.1		
-190G>T	G	100	100	99.5	0.39	0.28
	T	0	0	0.5		
-126T>C	T	97.7	95	98.5	0.7	0.21
	C	2.3	5	1.5		
-83G>A	G	88.2	93.7	87.8	0.9	0.1
	A	11.8	6.3	12.2		
c.250G>A (Arg76Lys)	G	89	95	89.7	0.87	0.1
	A	11	5	10.3		
c.499A>G (Leu159Leu)	A	100	97.5	100	1	0.08
	G	0	2.5	0		
c.520G>C (Leu166Leu)	G	100	98.8	100	1	0.28
	C	0	1.2	0		
c.877G>T (Thr285Thr)	G	100	100	99.5	0.47	0.28
	T	0	0	0.5		
c.997G>A (Thr325Thr)	G	99.1	98.8	99.5	1	0.48
	A	0.9	1.2	0.5		
c.1063T>C (Tyr347Tyr)	T	99.1	95	99	1	0.31
	C	0.9	5	1		
C.12145A>G (Lys398Arg)	A	100	100	99.5	0.47	0.28
	G	0	0	0.5		

The full description of -700_699ins is -700_699insCAGACACACATATACATGCACATACACA. In the table, I indicates the insertion allele, WT indicates the wild-type allele, and n is the total number of chromosomes. The asterisk indicates POAG versus controls and the sharp (hash mark) indicates OHT versus controls.

**Table 6:** Genotype frequencies of *myocilin* promoter and coding sequence variations in primary open-angle glaucoma, adult-onset ocular hypertension, and control subjects.

**Polymorphism**	**Allele1/Allele2**	**POAG (%) (n=110)**	**OHT (%) (n=40)**	**Control (%) (n=98)**	P*	**P#**
-1000C>G	C/C	80	82.5	86.7	0.26	0.12
	C/G	20	12.5	13.3		
	G/G	0	5	0		
-700_699ins#	WT/WT	100	97.5	100	1	0.28
	WT/I	0	2.5	0		
	I/I	0	0	0		
-387C>T	C/C	80	82.5	86.7	0.3	0.12
	C/T	19	12.5	13.3		
	T/T	1	5	0		
-339(GT)11-19	11/15	1.8	0	0	0.44	0.47
	12/13	18.2	33.3	30.6		
	12/14	1.8	0	1		
	12/15	0	0	1		
	13/14	24.5	10	22.5		
	13/15	23.6	33.3	27.5		
	13/16	1.8	0	1		
	13/19	0	3.3	0		
	14/15	27.3	20	15.3		
	15/16	0.9	0	1		
-315G>A	G/G	100	97.5	100		
	G/A	0	2.5	0		
	A/A	0	0	0		
-306G>A	G/G	64.5	65	64.3	0.6	0.74
	A/G	33.6	32.5	34.7		
	A/A	1.9	2.5	1		
-224 T>C	T/T	62.7	55	49	0.1	0.7
	C/T	34.5	32.5	41.8		
	C/C	2.7	12.5	9.2		
-190G>T	G/G	100	100	99	0.47	1
	G/T	0	0	1		
	T/T	0	0	0		
-126T>C	T/T	95.5	90	96.9	0.69	0.21
	C/T	4.5	10	3.1		
	C/C	0	0	0		
-83G>A	G/G	78.1	88.1	76.5	0.94	0.24
	A/G	21	11.9	22.5		
	A/A	0.9	0	1		
c.250G>A (Arg76Lys)	G/G	79	89	79.6	1	0.27
	A/G	20	11	19.3		
	A/A	0.9	0	1		
c.499A>G (Leu159Leu)	A/A	99	97.5	100	1	0.28
	A/G	1	2.5	0		
	G/G	0	0	0		
c.520G>C (Leu166Leu)	G/G	99	100	100	1	1
	G/C	1	0	0		
	C/C	0	0	0		
c.877G>T (Thr285Thr)	G/G	100	97.5	99	0.47	0.2
	G/T	0	2.5	1		
	T/T	0	0	0		
c.997G>A (Thr325Thr)	G/G	97	97.5	99	0.62	0.47
	G/A	3	2.5	1		
	A/A	0	0	0		
c.1063T>C (Tyr347Tyr)	T/T	96.3	95	98	0.68	0.32
	T/C	3.7	5	2		
	C/C	0	0	0		
C.1215A>G (Lys398Arg)	A/A	100	100	99	0.47	1
	A/G	0	0	1		
	G/G	0	0	0		

The full description of -700_699ins is -700_699insCAGACACACATATACATGCACATACACA. In the table, I indicates the insertion allele, WT indicates the wild-type allele, and n is the total number of chromosomes. The asterisk indicates POAG versus controls and the sharp (hash mark) indicates OHT versus controls.

In addition, nine sequence variations with MAFs greater than or equal to 5% were identified: -700_699ins, -315G>A, -190G>T, c.499A>G (Leu159Leu), c.520G>C (Leu166Leu), c.877G>T (Thr285Thr), c.997G>A (Thr325Thr), c.1063T>C (Tyr347Tyr), and c.1215A>G (Lys398Arg; Figure 1 and Table 5). The last SNP was detected only in a control subject (Table 5 and Table 6). To the best of our knowledge two of these SNPs (-315G>A and -190G>T) and the 28 bp insertion (-700_699insCAGACACACATATACATGCACATACACA) have not been previously described. They were found in two different OHT patients (-315G>A and -700_699ins) and in one control subject (-190G>T; Table 5 and Table 6). The 28 bp insertion was located in an AP1-like sequence (Figure 1). Of the remaining six rare polymorphisms, five were synonymous mutations (Leu159Leu, Leu166Leu, Thr285Thr, Thr325Thr, and Tyr347Tyr), while one (Lys398Arg) originated a conservative amino acid substitution (Figure 1). All of them except Leu166Leu have been previously reported [8,29]. Association analysis of these polymorphisms with the disease was limited by their low frequencies (Table 5 and Table 6).

### *Myocilin* linkage disequilibrium structure

**Figure 2:**
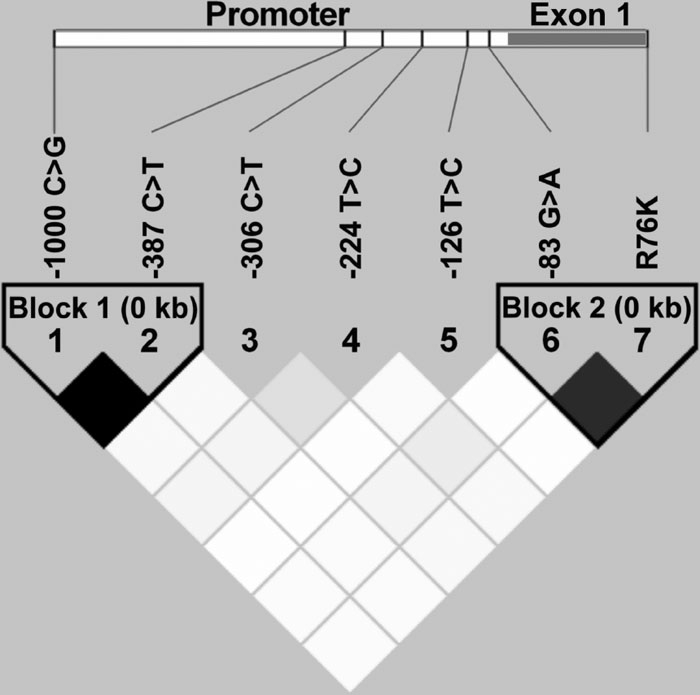
Pairwise linkage disequilibrium pattern of *myocilin* single nucleotide polymorphisms measured by D'. The location of each tested SNP along the *MYOC* gene is indicated at the top. The strength of LD is depicted by grey intensity, which moves from light grey to black as D' progresses from 0 to 1.

To determine the linkage disequilibrium (LD) structure of the *MYOC* gene in our population, we evaluated in the control group pairwise LD between all SNPs with MAF >5%. Two LD blocks were detected (Figure 2). Block 1 comprises SNPs -1000C>G and -387C>T (D'=1.0; D' confidence bounds=0.88-1.0) while block 2 is composed of SNPs -83G>A and Arg76Lys (D'=0.95; D' confidence bounds=0.80-0.99). The same LD structure was observed in glaucoma patients (data not shown).

### Haplotype analysis

*MYOC* SNPs with MAF >5% were used to construct predicted haplotypes, taking into account only one SNP from each LD block (-1000C >G from block 1 and -83G>A from block 2). Twenty haplotypes with frequencies >2% were inferred from our data, but only five exhibited frequencies >5% in the three groups (Table 7). The rare inferred haplotypes (<5%) were pooled in one class to allow comparison between cases and controls. We did not find any significant differences in predicted haplotype frequencies between cases and controls (Table 7), which indicates that they do not contribute to the development of glaucoma.

**Table 7:** Frequencies of *myocilin* inferred haplotypes in primary open-angle glaucoma, adult-onset ocular hypertension, and control subjects.

**Haplotype**	**POAG (%)**	**OHT (%)**	**Control (%)**	p*	**p#**
H9 (C-13-G-T-T-G)	19.3	21.1	21.1	0.055	0.83
H7 (C-13-G-C-T-G)	14.2	21.1	20.6		
H15 (C-15-A-T-T-G)	11.3	13.2	16.7		
H18 (G-15-G-T-T-G)	14.6	9.2	6.1		
H4 (C-12-G-T-T-G)	5.2	7.9	5.6		
Rest of haplotypes	35.4	27.6	30		

Haplotypes were constructed with polymorphisms, -1000, -339(GT)11-19, -306, -224, -126 and -83. Predicted haplotypes with frequencies lower than 2% were not considered. Only haplotypes with frequencies higher than 5% in all groups are shown. The asterisk indicates POAG versus controls and the sharp (hash mark) indicates OHT versus controls.

### Identification of *myocilin* pathogenic mutations in sporadic primary open-angle glaucoma cases

One non-sense (Gln368stop) and two missense (Ala445Val and Tyr479His) mutations were identified in three POAG patients (2.7%; Figure 1 and Table 8). All of them were present in heterozygosis and affected amino acid positions located in the olfactomedin-like domain (exon 3) of myocilin. Two of these mutations (Gln368Stop and Ala445Val) were previously reported in POAG [8,30] and as far as we know, the third mutation (Tyr479His) has been detected for the first time in the present study. Ages at diagnosis ranged from 32 to 56 years in this group of POAG patients (mean of 51.6 years; Table 8). In our sample, the mutation Gln368Stop (patient number 67) was associated with a severe phenotype featured by severe visual field alteration, high optic disk excavation, and resistance to medical treatment, which requires filtration surgery for an adequate control of IOP (Table 8). Carriers of mutations Ala445Val (patient number 50) and Tyr479His (patient number 3) showed early alteration of the visual field and their IOPs were adequately controlled with drugs (Table 8). The Tyr479His mutation was associated with an early-onset of the disease (32 years). Additionally, we also found the novel myocilin mutation Arg346Thr in patient number 19 who was diagnosed with glaucoma at 44 years and showed a narrow-angle (Table 8). Due to the narrow-angle, this patient was not included in the group of POAG subjects carrying *MYOC* mutations. After diagnosis, this subject underwent Nd:YAG laser iridotomy to prevent acute angle-closure glaucoma followed by treatment with three drugs (pilocarpine, dorzolamide, and timolol) to reduce IOP. In spite of this treatment, he required filtration surgery for the correct control of IOP. After 22 years of evolution, this patient displayed an extreme clinical phenotype characterized by bilateral and severe visual field alteration and large C/D ratios (Table 8).

**Table 8:** Clinical features of glaucoma patients with pathogenic *myocilin* mutations.

**Mutation**	**Subject number**	**Age at time of the study**	**Age at diagnosis**	**Gender**	**IOP OD/OS (mmHg)**	**C/D Ratio (OD/OS)**	**Visual field alteration (OD/OS)**	**Iridocorneal angle**	**Treatment (number of drugs)/surgery**
R346T* c.1059 G>C)	19	66	44	M	10/10	0.9/0.7	Severe/severe	1/2	3/Yes
Q368Stop c.1124C>T)	67	68	56	M	20/16	0.9/0.9	Severe/severe	4	2/Yes
A445V c.1356 C>T)	50	72	67	F	15/15	ND/ND	Early/early	4	2/No
Y479H* c.1457 T>C)	3	40	32	M	14/16	0.4/0.4	Early/early	4	1/No

Mutation information was based on the *MYOC* GenBank accession number: NM_000261. IOP values were obtained following medical treatment. IOP records before treatment were not always available. The asterisk indicates novel mutations. IOP: intraocular pressure, OD: right eye, OS: left eye, C/D: cup-disc ratio of optic nerve, ND: not determined due to oblique insertion of the optic nerve head.

### Evaluation of the two novel myocilin mutations pathogenicity by multiple sequence alignment and transient expression in 293T cells

**Figure 3:**
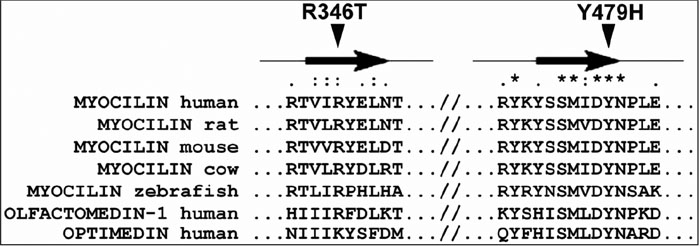
Multiple amino acid sequence alignment of myocilin from different species. Sequence alignment was generated by ClustalW. Residues affected by mutations are indicated by arrowheads. Asterisks indicate amino acid positions at which all query sequences are identical. Amino acid positions at which all analyzed sequences have amino acids that are chemically similar are denoted by two dots (:). One dot denotes amino acid positions with weak chemical similarity (.). Arrows indicated regions of the polypeptide chain which are predicted to fold into a beta-sheet conformation.

We used three approaches to evaluate the pathogenicity of the novel mutations: (a) analysis of evolutionary conservation of affected amino acids; (b) prediction of physicochemical changes induced by the different mutations; and (c) study of expression and subcellular distribution of cloned mutant and wild-type myocilin in transiently transfected 293T cells. Comparison of amino acid sequence alignment among myocilin from different species as well as with other members of the olfactomedin family of human proteins (olfactomedin-1 and optimedin) showed that the two novel mutations affected highly conserved amino acid residues (Arg346 and Tyr479), which are located in two regions of predicted beta-sheet folding (Figure 3). In addition, the two novel non-conservative mutations altered the predicted physicochemical properties of the polypeptide chain. The positive charge of Arg at position 346 is substituted by the polar Thr side chain in the mutant protein. Similarly, the hydrophobic Tyr is replaced by the polar His residue at amino acid position 479. These predicted amino acid changes could disrupt the secondary structure of myocilin, resulting in protein misfolding.

**Figure 4:**
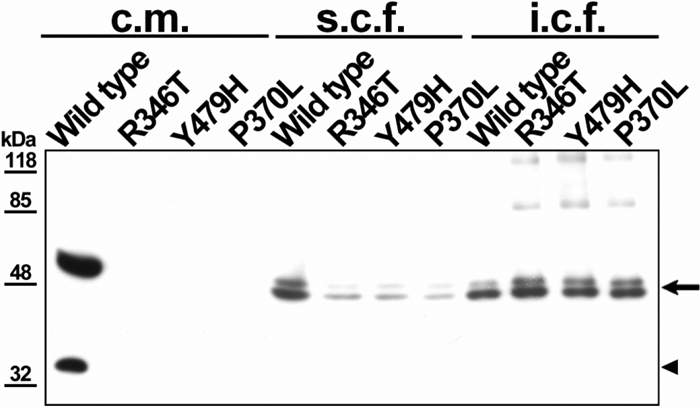
Western immunoblot of two novel myocilin mutations found in this study and expressed in transiently transfected 293T cells. Two hundred nanograms of DNA constructs encoding myc epitope-tagged versions of mutant myocilin forms (Arg346Thr, Tyr479His, Gln368Stop and Pro370Leu) were transfected into 293T cells. Separation of culture medium, soluble cellular fractions, and insoluble cellular fractions were carried out as indicated in the Materials and Methods. Detection was performed with an anti-myc monoclonal antibody. Myc-tagged wild-type myocilin was used as a control of normal expression and the myocilin mutation Pro370Leu was employed as a control of disease-causing mutation. The arrow and arrowhead indicate the position of the 55 kDa and 35 kDa myocilin bands, respectively. c.m.: culture medium; s.c.f.: soluble cellular fraction; i.c.f.: insoluble cellular fraction.

Transient expression of the two novel missense myocilin mutants in 293T cells showed that they accumulated intracellularly, mainly in the insoluble cellular fraction (Figure 4). The same behavior was observed with the myocilin mutation, Pro370Leu, which was used as a control because it is associated with one of the most severe myocilin glaucoma phenotypes [31]. A 35 kDa myocilin fragment was present in the culture medium of cells expressing wild-type myocilin (Figure 4, culture medium lanes), which is produced by proteolytic cleavage of the protein [25]. This fragment was neither detected in the two myocilin mutants nor in the control mutation, Pro370Leu (Figure 4), indicating that the proteolytic processing is reduced by these mutations, as previously described for myocilin pathogenic mutations [25].

**Figure 5:**
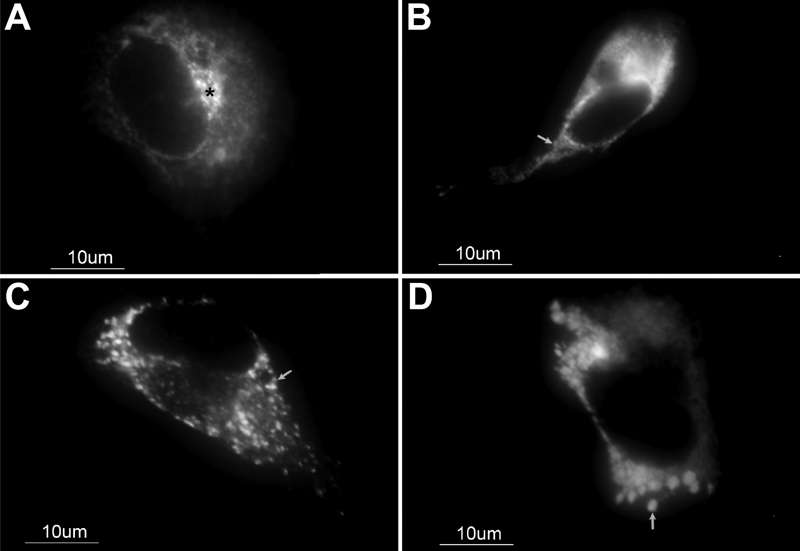
Subcellular distribution in transiently transfected 293T cells of human wild-type myocilin-GFP and two novel myocilin mutations found in this study. Two hundred nanograms of DNA constructs encoding wild-type myocilin (**A**), mutant myocilin forms, Arg346Thr (**B**) and Tyr479His (**C**), and the control, Pro370Leu (a disease causing mutation; **D**) were transfected into 293T cells. Wild-type myocilin was mainly detected in structures compatible with the Golgi apparatus and secretory vesicles. Note that the three mutant versions accumulated in the ER. The asterisk indicates the location of the Golgi apparatus. Arrows indicate the position of intracellular myocilin aggregates. Original magnification: X1600.

Immunocytochemical analysis of the two novel mutant myocilins transiently expressed in 293T cells revealed intense granular signals in the cytoplasm (Figure 5B and Figure 5C). This indicates most of the mutant myocilins accumulated intracellularly in the ER as misfolded proteins. This staining pattern clearly contrasted with that of wild-type myocilin, which was distributed in a reticular network located around the nucleus and cytoplasm and labeled a perinuclear structure compatible with the Golgi apparatus (Figure 5A). These results agree with previous reports [25,32-36] and strongly support that the two novel mutations found in the glaucoma patients are pathogenic.

### Analysis of *optineurin* sequence variations in sporadic cases of primary open-angle glaucoma

To evaluate the role of *OPTN* DNA sequence variations in Spanish patients affected by POAG, we screened the complete coding region of the gene in cases and controls by SSCP. Analysis by PCR DNA sequencing of the SSCP positive samples revealed two different G>A transitions, which originated two synonymous SNPs: Thr34Thr and Leu41Leu (Table 9). Both SNPs mapped to exon 4 and have been previously described in other populations [18,37-40]. Thr34Thr is a common polymorphism in our population, whereas Leu41Leu is a relatively rare one with MAFs of 1.8% and 2.6% in POAG and controls, respectively (Table 9). The low frequency allele (A) was not detected in the OHT group. Their genotype frequencies are shown in Table 10. There were no statistically significant differences in either allele or genotype frequencies between cases and controls (Table 8 and Table 9). These data indicate that *OPTN* DNA sequence variations are not involved in high-pressure POAG in the Spanish population.

**Table 9:** Allele frequencies of *optineurin* single nucleotide polymorphisms in primary open-angle glaucoma, adult-onset ocular hypertension, and control subjects.

**Polymorphism**	**Allele**	**POAG (%) (n=220)**	**OHT (%) (n=80)**	**Control (%) (n=196)**	p*	**p#**
c.412G>A (Thr34Thr)	G	66.4	75	65.3	0.82	0.11
	A	33.6	25	34.7		
c.433G>A (Leu41Leu)	G	98.2	100	97.4	0.74	0.32
	A	1.8	0	2.6		

A χ^2^ test or Fisher's exact test was used to compare allele frequencies in POAG or OHT patients with control subjects. Allele frequencies do not statistically differ between cases and controls. The asterisk indicates POAG versus controls and the sharp (hash mark) indicates OHT versus controls.

**Table 10:** Genotype frequencies of *optineurin* single nucleotide polymorphisms in primary open-angle glaucoma, adult-onset ocular hypertension, and control subjects.

**SNP**	**Allele1/Allele2**	**POAG (%) (n=110)**	**OHT (%) (n=40)**	**Control (%) (n=98)**	p*	**p#**
c.412G>A (Thr34Thr)	G/G	44.3	56.6	49.9	0.06	0.2
	G/A	44.3	36.6	31		
	A/A	11.4	6.6	19		
c.433G>A (Leu41Leu)	G/G	98.2	100	94.6	0.25	0.32
	G/A	1.8	0	5.3		
	A/A	0	0	0		

A χ^2^ test or Fisher's exact test was used to compare genotype frequencies in POAG or OHT patients with control subjects. Genotype frequencies do not statistically differ between cases and controls. The asterisk indicates POAG versus controls and the sharp (hash mark) indicates OHT versus controls.

## Discussion

Information regarding the role of *MYOC* and *OPTN* in Spanish POAG patients is scarce. So, the contribution of *OPTN* sequence variations to POAG in Spain has not been analyzed so far. Therefore, the main purpose of this study was to analyze the contribution of *MYOC* and *OPTN* sequence variations to adult-onset glaucoma in patients from this country.

We have found that heterozygous glaucoma *MYOC* mutations are located in the olfactomedin-like domain in 2.7% of POAG patients from Southeast Spain in accordance with frequencies reported in other populations [9,29]. One of the most interesting findings of this study was the identification of the novel mutation Tyr479His in an early-onset glaucoma patient with a mild phenotype. The high evolutionary conservation of the affected amino acid residue together with the biochemical and microscopy analysis supports the pathogenicity of this mutation. Two of the identified mutations, Gln368Stop and Ala445Val, have been previously described. Gln368Stop is the most common myocilin mutation found in POAG [8,29]. Interestingly, it is generally associated with late glaucoma onset (mean age at diagnosis 54.9 years) and low IOPs compared to other *MYOC* mutations [41]. Carriers of this mutation also show adequate responses to medical treatment similar to ordinary adult-onset POAG patients [8,9,29,30,41,42]. In contrast, our study found that Gln368Stop was associated with severe optic disk and visual damage and the patient who carried the mutation required surgery for a correct control of IOP. Since diagnosis was performed timely (at 56 years) further work is necessary to determine whether the phenotype is directly caused by this mutation or if it is influenced by other genetic and/or environmental factors.

Mutation Ala445Val has been previously found in OHT [43] and POAG patients from different populations [8,44]. The case subject who harboured this predicted amino acid substitution (number 50) showed a mild glaucoma phenotype. Noteworthy, a second novel mutation, Arg346Thr, was found in a patient with a narrow-angle. For this reason, it was not considered as a mutation found in POAG patients. Interestingly, this subject was diagnosed with glaucoma at 44 years of age, in contrast with typical closure-angle glaucoma which usually manifests at older ages. Preventive iridotomy to prevent pupillary block, followed by medical treatment with three drugs were not sufficient to reduce IOP thus required filtration surgery. These data indicate that the narrow angle is not the primary cause of glaucoma in this patient. Furthermore, it has been reported that myocilin mutations are not associated with angle-closure glaucoma, at least in Chinese patients [45]. Altogether, these data suggest that the narrow angle and the myocilin mutation could be coincidental in this patient and that Tyr479His could be involved in POAG development. Further investigations are required to determine the exact role of this mutation in POAG.

A previous study identified 7.5% of *MYOC* mutation carriers in patients from Galicia (N. Spain), but only sequence variations in exon 3 were analyzed [46]. Apart from Gln368Stop, which has also been identified by Vazquez and co-workers, the spectrum of pathogenic mutations was different from that found in the present study. It remains to be investigated whether these differences can be attributed to different genetic backgrounds between these two Spanish subpopulations or to the sample size used in the two studies. The same researchers later analyzed *MYOC* mutations in exons 1 and 2 and in the promoter region of this group of patients. No mutations in these two exons were found, and although five sequence variations were identified in the promoter region, no association with the disease was established [47], which agrees with our results.

In a previous study we found that approximately 10% of Spanish POAG patients carry mutations in the *CYP1B1* gene [17], which is three times higher than the frequency of carriers of mutations in the *MYOC* gene. This data clearly shows the existence of genetic heterogeneity among Spanish POAG patients and indicates that *CYP1B1* sequence alterations are the most important genetically known cause of POAG, at least in our population.

In the present study we have identified 15 *MYOC* SNPs, one polymorphic GT microsatellite, and one 28 bp insertion. All these DNA sequence variations were distributed along the promoter and coding region of the gene. To the best of our knowledge, three of these SNPs (-315G>A, -190G>T, and Leu166Leu) and the -700_699ins have been identified here for the first time. None of the polymorphic DNA sequence variations showed significant association with glaucoma. Albeit some of these promoter SNPs were located in putative regulatory promoter sequences, it remains to be demonstrated whether they affect *MYOC* expression. In any event, it is unlikely that changes in the gene expression may contribute to myocilin glaucoma since development of the disease appears to be related with structural alterations of the protein [34,48,49].

In accordance with previous reports, we found that allele and genotype frequencies of SNP -1000C>G were not significantly different in cases and controls [50-52]. Since Colomb et al reported the association of this SNP with the severity of POAG, there has been some controversy about the actual relationship with the disease. Our data support that there is no association between this polymorphism and the disease in Spanish patients.

We detected two LD blocks composed of SNPs -1000C>G and -387C>T (block 1) and -83G>A and Arg76Lys (block 2). LD block 1 has been described in the Chinese population [53] whereas LD block 2 has been found in Asian [53-58] and European populations [10]. Analysis of inferred six loci haplotypes further confirmed no association of *MYOC* promoter polymorphisms with either OHT or POAG in the studied Spanish population.

Defects in *OPTN* have been clearly implicated in normal tension glaucoma (NTG) [18,40], but its role in high-pressure glaucoma has been a source of controversy [59-61]. In accordance with previous reports, our data indicate that *OPTN* does not contribute to the development of either OHT or typical adult-onset high-pressure glaucoma, at least in the Spanish population [62].

The present study provides new insight into the role of *MYOC* and *OPTN* genes in POAG in Spain and brings new information to unravel genetic alterations associated with POAG in this country.
